# Anastomotic dehiscence (AD) in colorectal cancer surgery: mechanical anastomosis versus manual anastomosis

**Published:** 2012-12-25

**Authors:** C Oprescu, M Beuran, AE Nicolau, I Negoi, MD Venter, S Morteanu, AM Oprescu-Macovei

**Affiliations:** * General Surgery Department, Emergency Hospital of Bucharest, Romania; ** Gastroenterology Department, Emergency Hospital of Bucharest, Romania

**Keywords:** colorectal cancer, mechanical suture, anastomotic dehiscence

## Abstract

Introduction: Anastomotic dehiscence (AD) is the “Achilles heel" for resectional colorectal pathology and is the most common cause of postoperative morbidity and mortality. AD incidence is 3-8%; mortality rate due to AD two decades ago was around 60% and at present is 10% [**4-6**]. This paper analyzes the incidence of AD after colorectal resection performed both in emergency and elective situations, depending on the way it is done: manually or mechanically.

Methods: Retrospective, single-center, observational study of patients operated in the period from 1st of January 2009 to 31th of December 2011 for malignant colorectal pathology in the Emergency Clinical Hospital of Bucharest.

We evaluated the incidence of digestive fistulas according to the segment of digestive tract and time from hospital admission, to the way the anastomosis was achieved (mechanical vs. Manual), to the complexity of intervention, to the transfusion requirements pre/intra or postoperative, to the past medical history of patients (presence of colorectal inflammatory diseases: ulcerative colitis and Crohn's disease), to the average length of hospital stay and time of postoperative resumption of bowel transit.

Results: We included 714 patients who had surgery between 1st of January 2009 and 31th of December 2011. 15.26% (109/714) of the cases were operated in emergency conditions. Of the 112 cases of medium and lower rectum, 76 have “benefited" from preoperative radiotherapy with a fistula rate of 22.36% (17/76). The incidence of anastomotic dehiscence in the group with preoperative radiotherapy and mechanical anastomosis was 64.7% (11/17) versus 35.3% (6/17) incidence recorded in the group with manual anastomosis.

Colorectal inflammatory diseases have been found as a history of pathology in 41 patients - incidence of fistulas in this group was of 12.2% (5/41), compared to only 6.83% (46/673) incidence seen in patients without a history of such disease. For the group with bowel inflammatory disease, anastomotic dehiscence incidence was of 13.8% (4/29) when using mechanical suture and 8.3% (1/12) when using manual suturing.

The period required for postoperative resumption of intestinal transit was of 3.12 days for mechanical suturing and 3.93 days in case of manual suture.

The mean time (MT) to perform the ileocolic and colocolic mechanical anastomosis is 9 ± 2 minutes. If anastomosis is “cured" with surjet wire or separate threads, MT is 11 ± 5 minutes. MT to perform the ileocolic and colocolic manual anastomosis is 9 ± 3 minutes for surjet wire and 18 ± 5 minutes for separate threads. MT to perform the colorectal mechanical anastomosis is 15 ± 4 minutes. MT to perform the colorectal manual anastomosis is 30 ± 7 minutes (using separate threads).

Detailing the nature of the surgical reinterventions, we have found: 7 reinterventions for AD post mechanical anastomoses (1 case of suture defect, 2 cases of resection and re-anastomoses, 4 cases with external branching stoma); 5 reinterventions for AD post manual anastomoses (0 cases of suture defect, 1 case of resection with re-anastomosis, 4 cases of external shunt stoma).

In the analyzed group, we recorded a total of 57 deaths from a total of 714 cases resulting in a mortality rate of 7.98%.

Conclusions: Mechanical suture technique is not ideal for making digestive sutures.

With the exception of low colorectal anastomoses where mechanical sutures are preferable, we cannot claim the superiority of mechanical anastomoses over those manually made, for colorectal neoplasia.

## Introduction

Currently, colorectal cancer in men is ranked third worldwide, after lung and prostate cancer and second, in women, after breast cancer, with a men to women ratio of 1.5. Also, for the first time cancer has surpassed heart disease [**1,2,4**].

In our country, the incidence of colorectal cancer is still increasing (from 8.78/10000 inhabitants in 1998 to 23.79/10000 inhabitants in 2008) [**3,4**].

In the last decades, several types of anastomoses were designed and introduced into practice to prevent anastomotic dehiscence: mechanical stapler, the telescoping anastomosis, bio-fragmented ring anastomosis [**1-3**]. 

Anastomotic dehiscence is the “Achilles heel" for postoperative colorectal pathology and it is the most common cause of postoperative morbidity and mortality. Its general incidence is 3-8%. The mortality rate due to AD was around 60% two decades ago and is 10% at present [**4-6**].

Attempts to create a mechanical system to achieve digestive anastomoses dates back to the late nineteenth century with the creation of John Benjamin Murphy’s “anastomotic button". Modern age of mechanical sutures began in 1956 at the Institute for Scientific Apparatus and Experimental Surgical Instruments in Moscow where there were made virtually all types of staplers that, in one form or another, are used today: linear suture stapler for linear anastomosis and circular suture stapler [**10-14**]. 

This paper analyzes the incidence of AD after colorectal resection performed during emergency and elective surgery in both manually or mechanically anastomosis techniques.

## Methods

Retrospective, single-center, observational study of patients operated in the period of 1st of January 2009 – 31th of December 2011 for malignant colorectal pathology at The Emergency Clinical Hospital of Bucharest was realized.

The evaluation was based on clinical history, physical examination, biological samples analysis, morphological explorations, data reported intraoperatively correlated to those observed in the interventions that we have performed, pathological bulletins and clinical observations on the evolution of such cases. Only cases of histologically confirmed colorectal cancer were included. All cases operated laparoscopically were excluded.

We considered as complex surgical resections those interventions that associate to colon/rectal site an intervention in the urology, gynecology or upper digestive tract site.

The presence of fistula is reasoned, in any case, clinically – presence of gas / enteral content on drainage tube, using medical imaging and visually - if reintervention is performed.

Anastomoses that were included in the study: ileocolonic, colocolonic, colorectal anastomoses. We also included Hartmann operations for rectal stump fistula. The reintegration interventions and amputations of the rectum were included in the study.

We evaluated the incidence of digestive fistulas according to the segment of digestive tract involved and the time from hospital admission, to the way the anastomosis (mechanical / manual) was achieved, to surgical intervention complexity, to the transfusion requirements pre / intra / postoperatively, to past medical history of patients (presence of colorectal inflammatory diseases: ulcerative colitis and Crohn's disease), to the average length of hospitalization, to postoperative time to the resumption of bowel transit.

To centralize data, the recto-sigmoid junction cancers were assimilated to rectal cancer for the anastomosis is also colorectal; we also included coloanal anastomoses performed for lower rectal neoplasm.

Statistical processing was performed through the EPIINFO 5 software; statistical estimation of the results was performed for a minimum acceptable threshold of significance in biology: p = 0.05, corresponding to a statistical accuracy of 95%, using criteria for decision of statistical tests (p <0, 05 = difference is significant).

## Results

We included 714 patients who had surgery between 1st of January 2009 and 31th of December 2011. 15.26% (109/714) of cases are emergency interventions (<24 hours after admission). We registered 31 interventions for complications of peritonitis and 55 interventions for complications of bowel occlusion (**Table 1**, **Table 2**, **Table 3**).

**Table 1:**
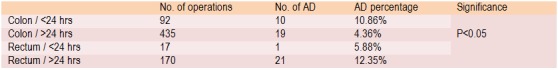
AD incidence and digestive segment implied according to time of admission

**Table 2:**
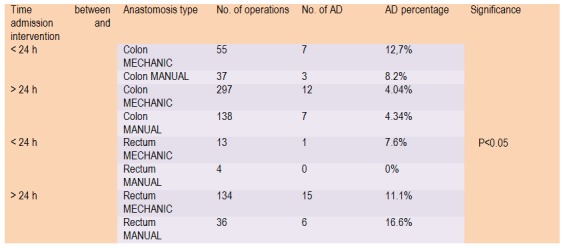
AD incidence and digestive segment implied according to time of admission

**Table 3:**
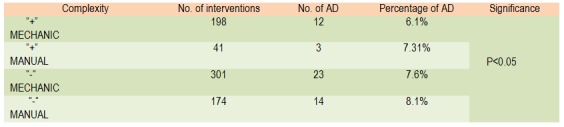
Distribution of AD depending on the complexity of surgery

Of the 112 cases of medium and lower rectum tumors 76 have "benefited" from preoperative radiotherapy with a fistula rate of 22.36% (17/76); in the group without preoperative radiotherapy fistula rate is of 14% (5/36). The incidence of anastomotic dehiscence in the group with preoperative radiotherapy and mechanical anastomosis is of 64.7% (11/17) versus 35.3% (6/17) incidence recorded in the group with manual anastomosis (**Table 4**, **Table 5**).

**Table 4:**
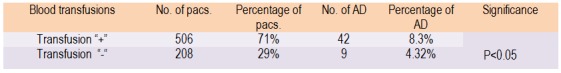
Fistulas rate distribution function of presence of blood transfusions

**Table 5:**
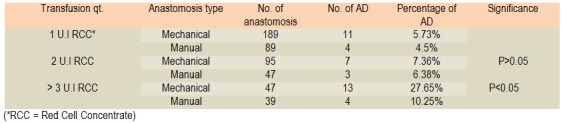
Fistulas distribution according to transfusion need and type of anastomosis

Colorectal inflammatory diseases (ulcerative colitis and Crohn's disease) have been found as pathological medical history in 41 patients; incidence of fistulas in this group is of 12.2% (5/41), compared to only 6.83% (46/673) incidence seen in patients without a history of such diseases. For the group with colorectal inflammatory diseases, the anastomotic dehiscence incidence is of 13.8% (4/29) when using mechanical suture instead of 8.3% (1/12) if using manual suturing.

Required postoperative period of time for the resumption of intestinal transit was of 3.12 days for the mechanical suturing and 3.93 days for the manual suturing.

When analyzing the anastomoses types that were performed we have found: ileocolic and colocolic anastomosis: mechanical: 52+297 = 349 anastomoses; manual: 35+138 = 173 anastomoses; colorectal anastomoses: mechanical: 9+134 = 143 anastomoses; Manual: 1+ 36 = 37 anastomoses.

The mean time (MT) to perform the ileocolic and colocolic mechanical anastomosis is 9 ± 2 minutes. If it is then "cured" with surjet wire or separate threads, the time is 11 ± 5 minutes. MT to perform the ileocolic and colocolic manual anastomosis: surjet wire 9 ± 3 minutes / separate threads 18 ± 5 minutes. MT to perform the colorectal mechanical anastomosis is 15 ± 4 minutes. MT to perform the colorectal manual anastomosis: with separate threads is 30 ± 7 minutes.

All anastomoses were performed without protective stoma.

Cost analysis showed:

- Mechanical sutures: minimum 2 staplers from which one is circular.

- Manual sutures: 5 silk threads no. 3.0 or prolen no. 3.0 with atraumatic needle.

Without detailing the prices, the cost difference is at least 8 times higher with mechanical sutures.

Postoperative evolution (**Table 6**): 653 patients with resection + anastomosis - surgical cure "per primam"; 12 patients with Hartmann type surgery - 2 patients in this group developed distal stump AD after mechanical suture; 49 patients with dehiscent anastomosis.

**Table 6:**
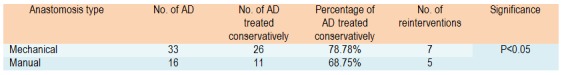
AD treatment

While detailing the surgical reintervention we have found: 7 reinterventions for AD post mechanical anastomoses: suture of defect - 1 case; 2 cases of resection and reanastomosis; external branching with stoma - 4 cases. 5 reintervention for AD post manual anastomoses: suture of defect - 0 cases; resection with reanastomosis- 1 case; external branching and stoma - 4 cases.

During the postoperative evolution, we analyzed immediate wound complications and we found the following (**Table 7**):

**Table 7:**
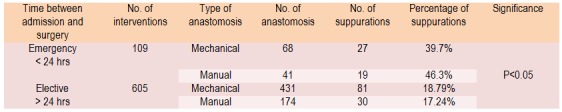
Distribution of wound suppuration

 Average length of hospital stay for patients who did not develop dehiscent anastomosis was of 8.07 days when using mechanical suture and 8.73 days for manual suture, no significant difference resulting.

 In the analyzed group, we recorded a total of 57 deaths from a total of 714 cases, resulting in a mortality rate of 7.98% (**Table 8**, **Table 9**, **Table 10**).

**Table 8:**
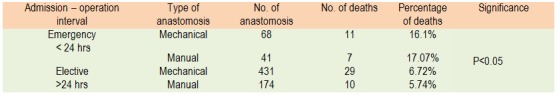
Distribution of deaths according to the interval between admission and operation

**Table 9:**
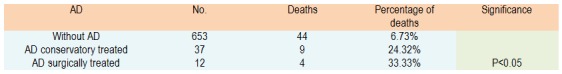
Distribution of deaths according to the presence of AD

**Table 10:**
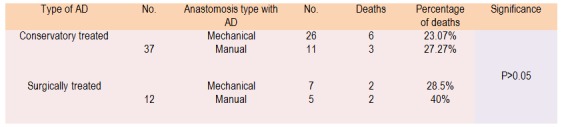
Distribution of deaths according to the type of anastomosis and to the presence of AD

## Discussion

Despite the progress in medicine, anastomotic fistula remains an issue of major concern for all surgeons. With the advent of mechanical sutures, many of us believed that digestive fistulas will not create as many problems as before, but is it so? Have we been excited too soon?

Parallel analysis of classical vs. mechanical techniques starts from the need to diminish or eliminate anastomotic failure.

AD may be complicated by peritonitis and septic shock if not identified and treated properly. It is due to the existence of global and local factors. Local factors include the following: the presence of infection in the surgical field, the creation of anastomoses with mechanical tension, reduced blood supply to the ends of the suture, presence of stenosis distally from anastomosis. General factors are: hypoproteinemia, vitamin D deficiency, uraemia, steroids treatment, diabetes mellitus, anaemia [**15-17**]. Colon wall is thin, vascularized according to the arteries of terminal type and it contains septic fluid, so anastomoses performed on colon are less reliable than those performed on stomach [**7-9**]. 

AD may be revealed through: elimination of stercoral fluid through the drainage tube, elimination of stercoral liquid through the wound, hernia, signs of generalized peritonitis, signs of sepsis without participation of abdominal signs (low reactivity) [**18,19**]. 

Postoperative intestinal atony, ileus, and vomiting cause an increase in pressure in the intestinal lumen. An anastomosis is more reliable as it is more resistant to increased intraluminal pressure.

All patients presented during the period of time before the development of fistulae states low grade fever, fever, prolonged postoperative ileus, events whose intensity decreases with externalizing fistulous fluid on tubes [**15,16,20**]. 

Anastomoses performed on colon during the emergency conditions produce fistulae which are more common then the rectum anastomoses (10.86%); also, while analyzing the type of suture used we can see that mechanical anastomoses are more likely to induce fistula occurrence then the manual ones (12.7% versus 8.2%). In terms of performing anastomoses electively, the colonic fistulas occur with the same frequency (4.04% versus 4.34%) regardless of the way they are made: mechanical or manual.

For a long time, the incidence of dehiscence of anastomoses performed in emergency conditions was justified by an insufficient mechanical preparation of the colon, this theory was revised with the introduction of the concept of "fast track surgery" - this concept combines preoperative patient education, new techniques anaesthetics, analgesics and surgery aimed at reducing the body's response to surgical stress, alleviate pain and discomfort, aggressive postoperative rehabilitation, including early enteral nutrition and rapid mobilization, also including a modern attitude in terms of general principles of postoperative care (using wells, drains, catheters) - fast track surgery may shorten postoperative recovery time, reduce the period of hospitalization and convalescence, and decreasing postoperative morbidity and mortality [**21-24**]. 

For anastomoses to the rectum, in operations performed in scheduled mode, fistulization recorded the same rate, regardless of the way they were made (manually or mechanically, 16.6%, 11.1%). We cannot say the same for those performed during the emergency surgery because Hartmann operations were preferred. However, without a statistical value, we have had two fistulas of rectal stump performed in emergency conditions, both after a mechanical suture.

Besides colonic / rectal time, in 239 cases it took other concomitant surgery: nephrectomy, gastric resections, enterectomias, anexectomies, colpectomies posterior partial cystectomy - surgery regardless of the extent or manner in which mechanical / manual AD occur with the same frequency of 6.1% and 7.31%.

Preoperative Radiotherapy destroys cancer cells but also has adverse effects on adjacent normal tissues and causes decreased blood flow with this effect due to fibrosis and stenosis of blood vessels [**25,26**]. The preoperative radiotherapy is certainly a predisposing factor for the occurrence of anastomotic fistula – meeting it in 22.36% of the cases compared to 14% as it occurs in cases without radiotherapy. The anastomotic type influences the anastomosis fistula rate as well - mechanical sutures on irradiated rectum fail more frequently than manual sutures (64.7% versus 35.3%).

The impact of anaemia on digestive anastomoses is a widely debated topic. There are authors who argue that anaemia has no role in the development of fistula as an independent factor, but only in the presence of hypovolemia may cause tissue hypoxia favouring dehiscence. However, in the present study, the presence of anaemia favours the occurrence of fistula - patients requiring blood transfusion pre, intra and/or postoperative recorded incidence of anastomotic fistula of 8.3% compared to 4.32% for patients where correction of anaemia was not necessary. Analyzing the type of anastomosis we can say that fistula incidence is greater with mechanical sutures vs the manual ones in the presence of anaemia [**27-29**]. 

The role of the Inflammatory Bowel Disease (Crohn's disease and ulcerative colitis), as systemic factor affecting digestive anastomosis healing, has not been well defined until now. Digestive fistulas still occur in a proportion of 12.2% in patients having a history of these diseases, a much higher percentage than the one of the control group - 7.14%. In the presence of Crohn's disease and ulcerative colitis, the mechanical sutures most frequently fail (13.8%) vs. manual sutures (8.3%).

One common parameter analyzed during medical care is the period of hospitalization because it is directly proportional to the costs of the case. In order to be as objective as we can about the analyzed hospitalization days, we excluded patients who developed intestinal fistulae. Patients who received mechanical suture were discharged to 8.07 days after surgery and those who underwent manual suture at 8.73 days. There is no difference between the duration of hospitalization between patients with mechanical suture or those with a manual one.

Although resumption of intestinal transit is a parameter that depends on systemic factors, we discussed it in terms of the way anastomosis was performed. Resumption of bowel transit was considered the postoperative day when the patient presented gas emissions or had spontaneously defecation. If the mechanical bowel anastomoses are performed, bowel transit resumes at 3.12 days versus 3.93 days for the manual anastomosis (P> 0.05, not statistically significant). Even if there is a difference of about 20 hours, this cannot support the superiority of any anastomosis type [**10,17,20**]. 

Intraoperative duration has a direct reflection on postoperative evolution; the more the operating time is reduced the lower the frequency of postoperative complications is achieved. In this study, the rectosigmoid junction cancers were treated as rectal cancer because the anastomosis is also colorectal. The average time to perform ileocolic mechanical anastomosis and colocolic mechanical anastomosis is 9 ± 2 minutes and if anastomosis is "cured" with a separate wired or a wire surjet, the average time is 11 ± 5 minutes for the mechanical version; with wire surjet we recorded 10 ± 3 minutes and if you prefer the manual version with separate threads, 18 ± 5 minutes can be reached to perform the anastomosis in manual mode. For ileocolic anastomoses and colocolic mechanical sutures, the intraoperative time is not significantly shorted and in the hands of an experienced surgeon, sutures with surjet wire are even "faster" than the mechanical ones. The situation is much different for the colorectal anastomoses; this time making the mechanical anastomosis taking 15 ± 4 minutes vs. 30 ± 7 minutes for the manual variant. The most significant time savings are achieved when using the EEA stapler type, especially for the middle or lower rectum [**30-35**]. 

Cost per anastomosis is another parameter analyzed. For ethical reasons we cannot publish cash value of the materials used but we can conclude as it follows: for mechanical suturing at least two staplers are needed, including a circular one and a manual anastomosis which can be performed with an average of 5 3.0 silk wires with atraumatic needles. The difference in cost would be 8 times higher for mechanical sutures.

The main criteria behind the conservative treatment of anastomotic failures were the following: low flow (<250 ml / day), no signs of peritoneal irritation, surgical tactics - surgeon preference / experience.

Analyzing the evolution of postoperative digestive fistulae, we found that after manual suture, failures are with a higher flow and more need for a number of reinterventions compared to mechanical suture (31.25% manual versus 21.22% mechanical).

Parietal suppuration is more frequently met when performing emergency surgery compared with elective interventions; this set of data are not influenced by the manner in which the mechanical or manual anastomosis were performed (39.7%, 18.79% versus 46.3%, 17.24%).

Mortality in the analyzed group is of 7.98%, a value which is similar to that found in other studies: F. Merad - 14.3%, Rose D. - 5.9%, Mileski WJ - 25%, Alves A - 13% [20,36-40]. For the emergency cases operated, mortality is higher than that of those operated electively, and not influenced by the manner in which the anastomosis was performed: mechanically (16.1% versus 6.72%) or manually (17.07% versus 5.74%). Postoperative digestive fistula as a complication affects the mortality rate: anastomosis without fistulas - 6.73% mortality, fistula treated conservatively - 24.32% mortality, fistulas with reintervention - 33.33% mortality, values that are independent of the manner in which the anastomosis was performed: mechanically (23.07% versus 28.5%) or manually (27.27% versus 40%).

Without being able to obtain a statistical value we found a higher anastomotic bleeding rate from anastomosis made mechanically - one can explain this by Staples shape – shape of a “B" – when closed they do not occlude the vasculature so do not realize hemostasis. At 5 months postoperatively 3 patients who had surgery for cancer of the rectum using mechanical anastomosis were re-hospitalised for anastomotic stenosis; in 2 of them stricture was felt by the examining finger expansion being performed manually; the 3rd patient experienced a high stricture - 10 cm from the anus – re-expansion being made with the Hegar dilatator [**41-44**]. 

Limitations of the study are determined by the retrospective selection of the cases plus heterogeneity of the surgical team’s experience - operations were performed by a number of surgeons.

## Conclusions

Mechanical suture technique is not ideal for making digestive sutures.

 With the exception of low colorectal anastomoses, where mechanical sutures are preferable, mechanical anastomoses cannot claim superiority to those made manually, for colorectal neoplasia.

 AD is more common after operations performed as emergencies.

 Mechanical anastomoses most likely result in AD than those made manually, during interventions in emergency situations.

Acknowlegements

This paper is partially supported by the Sectoral Operational Programme Human Resources Development, financed from the European Social Fund and by the Romanian Government under the contract number POSDRU/88/1.5/S/64331.
